# The challenge of antimicrobial resistance in the Asia-Pacific: a pediatric perspective

**DOI:** 10.1097/MOP.0000000000001437

**Published:** 2025-02-04

**Authors:** Nguyen Xuan Huong, Michelle Harrison, Erena Kasahara, Ben Marais, Nina Dwi Putri, Phoebe CM Williams

**Affiliations:** a Phan Chau Trinh University, Vietnam; b Sydney Infectious Diseases Institute, University of Sydney, Sydney, Australia; c Philippine General Hospital, Manila, The Philippines; d Cipto Mangunkusumo Hospital, Jakarta, Indonesia; e Sydney Children's Hospital, Randwick, NSW; f School of Public Health, Faculty of Medicine, The University of Sydney; g School of Women and Children's Health, UNSW, Sydney, Australia

**Keywords:** antimicrobial resistance, child health, infant health, neonatal sepsis, pediatrics

## Abstract

**Purpose of review:**

The densely populated Asia Pacific region is home to 600 million children, and suffers from a significant burden of morbidity and mortality due to infections associated with antimicrobial resistance (AMR). We aimed to identify the drivers, challenges and potential opportunities to alter the burden of AMR within the region.

**Recent findings:**

Despite the high AMR burden borne by the Asia Pacific region, there are limited (and geographically imbalanced) published data to delineate the contemporary epidemiology of serious multidrug-resistant bacterial infections in children. Furthermore, the region is impacted by overcrowded and poorly resourced healthcare facilities, insufficient microbiological resources, and widespread community and environmental antibiotic use leading to limited efficacy for frequently prescribed antibiotics. Vaccine coverage is also inadequate and inequitable, further driving the burden of infectious disease (and antibiotic overuse) in children.

**Summary of implications:**

There are many challenges in implementing antimicrobial stewardship and infection prevention and control programs to reduce the excessive AMR disease burden in children across the Asia Pacific region, yet locally-driven strategies have successfully reduced antibiotic overuse in some settings, and should be replicated. Reducing the AMR disease burden will require improved healthcare resourcing, including better access to microbiological diagnosis, and multidisciplinary approaches to enhance infection prevention and antibiotic prescribing.

## INTRODUCTION

Infections due to antimicrobial resistance (AMR) are a pressing global health threat, responsible for more than one million deaths each year [1]. One in five deaths due to AMR occurs in children, largely due to preventable or treatable infections [2]. AMR affects all world regions, but is propagated by poverty – consequently, low- and middle-income countries are most significantly affected by the growing burden of AMR [3]. 

**Box 1:**
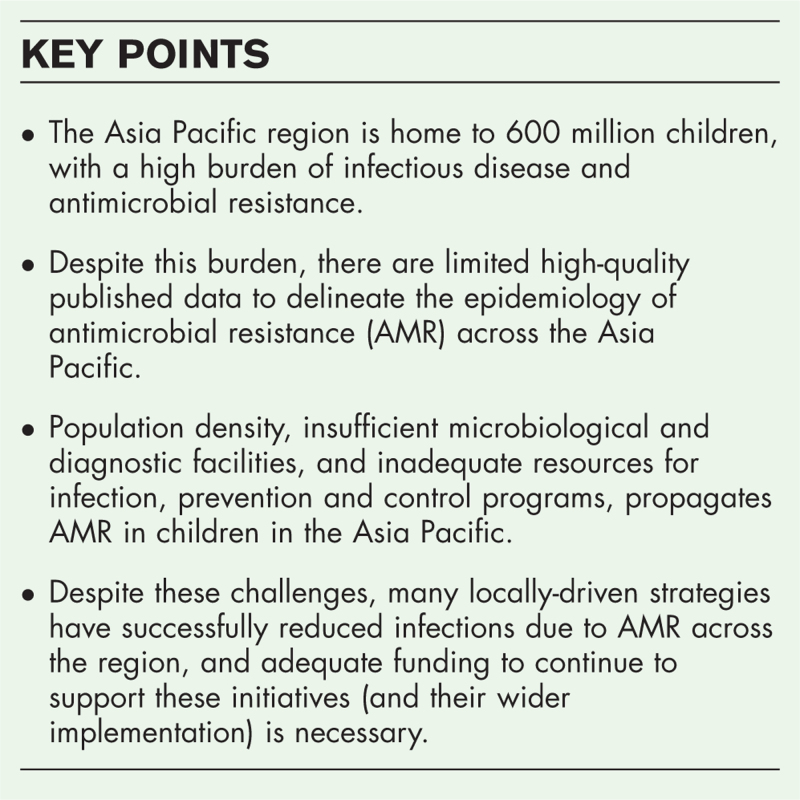
no caption available

**FIGURE 1:**
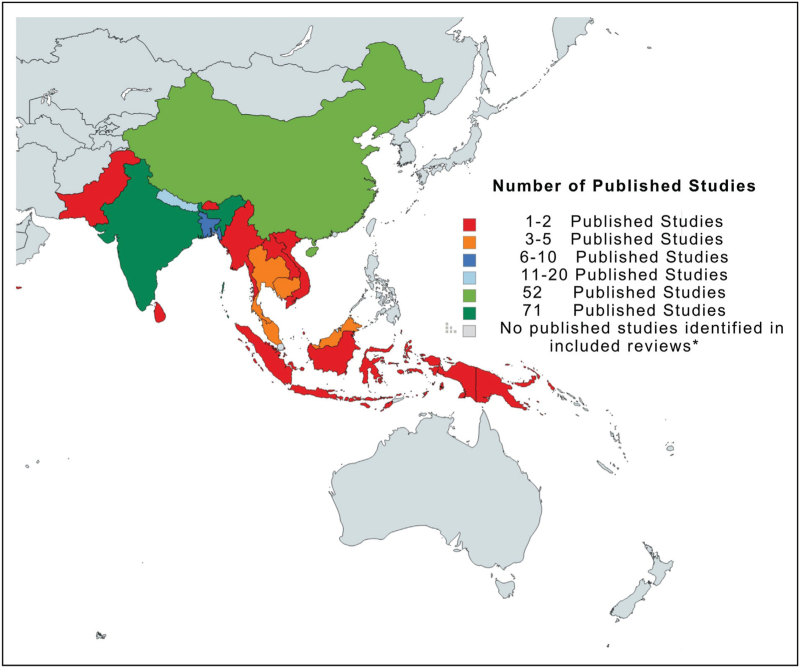
There are limited and geographically imbalanced published data to inform an accurate understanding of the true burden of AMR across the Asia Pacific region. AMR, antimicrobial resistance.

The Asia Pacific (comprising both Southeast Asia and the Western Pacific region) is home to 53% of the world's population, including > 600 million children [4]. Within the region, overcrowded healthcare facilities, limited access to clean water and sanitation, excessive and poorly targeted antibiotic use, and insufficient infection prevention and control (IPC) resources propagate the rapid transmission of multidrug-resistant pathogens [5]. Despite these challenges, there are limited high-quality regional data to inform an accurate understanding of the true burden of morbidity and mortality due to AMR across the Asia Pacific region (Fig. 1) [6^▪^]. However, the sparse and geographically imbalanced data that are available indicate that first-line antibiotics used to treat common childhood infections are increasingly inefficacious across the Asia Pacific, resulting in unnecessary deaths in infants and children [7^▪^].

The Asia Pacific region's high population density poses challenges in adequately resourcing healthcare systems to care for an increasing number of complex patients. This is particularly true for neonates, given the region's very high rates of premature delivery [8]. Geographic vulnerability to extreme weather events and rising vaccine hesitancy are additional factors that complicate care delivery in a modern world challenged by climate change and misinformation. In this review, we identify challenges and opportunities to address this rising burden of AMR on child health in the Asia Pacific.

### The challenge of diagnosing and managing infections in children in the Asia Pacific

Young children are highly vulnerable to infectious diseases, yet the accurate microbiological diagnosis of pediatric infections is challenging. Across all healthcare settings globally, there are difficulties in obtaining adequate blood culture sample volumes in neonates and children, which reduces the sensitivity of blood culture diagnostics. The reduced sensitivity of blood cultures in neonates and children is further compounded by the widespread community and peripartum use of antibiotics [9,10]. Consequently, there are high rates of ‘culture-negative’ infections that occur in neonates and children, often treated with broad-spectrum empiric antibiotics, that propagate the selection and spread of AMR [11]. In the Asia Pacific region, these challenges are further heightened by insufficient access to adequate microbiological facilities, including scarce highly-trained laboratory staff, and limited funding for accurate microbiological diagnosis and AMR surveillance [12,13].

Pathogen-specific diagnosis is key to ensuring targeted treatment can ensure optimal patient outcomes, alongside enabling the development of antimicrobial stewardship (AMS) programs to reduce the overuse of inappropriate and ineffective antibiotics [10]. However alongside these microbiological diagnostic challenges, there are also inconsistent syndromic definitions in children, further reducing the diagnostic accuracy of severe infections – particularly for neonates and infants. While sepsis definitions have been developed for adult and populations, often these are not applicable to low-resource settings [14], and there is no current international consensus definition for neonatal sepsis [15]. This further contributes to high rates of empiric antibiotic prescribing to cover for ‘suspected’ or ‘probable’ sepsis, particularly in preterm neonates, who are especially vulnerable to sepsis events during their prolonged hospitalization period [16].

These challenges combine to result in significant overuse of antibiotics in hospitalized children across the Asia Pacific, particularly in neonatal intensive care units, where increasing rates of multidrug-resistant infections are responsible for rising neonatal mortality [17]. In the context of rapidly rising AMR, it is clear that empiric guidelines to treat neonatal and pediatric infections quickly become outdated and require regular review and revision [16]. For example, The World Health Organization (WHO) currently recommends ampicillin/benzylpenicillin and gentamicin (or third-generation cephalosporins) for treating neonatal sepsis [18], however, recent observational studies have revealed a high proportion of both early- and late-onset neonatal infections are caused by gram-negative bacteria harbouring extended-spectrum beta-lactamases (ESBLs) and carbapenem-resistance mechanisms [19,20], rendering these recommended treatment regimens inefficacious [7^▪^,^13^,^21^,^22^].

The limited surveillance data available from the Asia Pacific region suggests that broad-spectrum antibiotics (such as carbapenems) remain somewhat efficacious in treating serious childhood infections [13], but there is widespread overuse of these agents which is already causing rising rates of carbapenem-resistant infections. Concerningly, a recent multicentre point prevalence survey conducted across multiple neonatal intensive care units in the Asia Pacific revealed very high prescribing rates of carbapenems and other WHO classified ‘Watch’ and ‘Reserve’ antibiotics [23], and research from Thailand reveals meropenem is the most commonly-prescribed antimicrobial in paediatric intensive care units [9].

Overuse of broad-spectrum antibiotics will result in a rising prevalence of infections that are increasingly difficult to treat, which is a major concern in light of the vastly inadequate antibiotic research and development pipeline, particularly for paediatric populations [24]. Delays in the licensing of new antibiotics for children [25], and a sparse clinical trial landscape to optimize the management of multidrug-resistant infections drive the currently high rates of prescribing of divergent and off-label antibiotics to treat serious infections in children across the Asia Pacific region [25].

### The role of vaccination in preventing antimicrobial resistance across the Asia Pacific Region

The development of vaccines has resulted in a historical decline in infectious diseases, enabling the prevention of six million deaths from vaccine-preventable diseases each year [26]. However, there are major disparities in vaccine coverage between high-income and low-income countries [27]. This inequity is particularly apparent for some of the more recently introduced vaccines: for example, although 65% of children globally have received three doses of the pneumococcal conjugate vaccine (reducing their risk of pneumonia, meningitis and bloodstream infections), only 26% of children in the Western Pacific region have received a pneumococcal conjugate vaccine [28].

Furthermore, in 2023, 14.5 million children were identified as “zero-dose children” – these are children who have not received any immunizations – a number that has risen significantly due to the impact of the COVID-19 pandemic on routine child healthcare and vaccine uptake [29]. In the Asia Pacific region, 3.4 million children missed routine immunizations due to the COVID-19 pandemic [30], which impeded not only vaccine delivery and routine immunization programs, but also heightened vaccine hesitancy and antivaccination sentiments [28].

Factors contributing to vaccine hesitancy are multifaceted, with complex sociocultural drivers; but ultimately vaccine hesitancy – which is rising across the Asia Pacific region - results in suboptimal immunization coverage and the emergence, or re-emergence, of vaccine-preventable diseases [31,32]. In the Philippines, the deaths of 14 children during a campaign for a new dengue vaccine weakened regional confidence in immunization and increased public mistrust in health experts. Recent declines in vaccine coverage have resulted in large measles and pertussis outbreaks across the country [33].

A rise in vaccine-preventable diseases perpetuates the AMR challenge, by increasing the provision of empiric antibiotics for children with infections (and often, also, their symptomatic contacts) [34]. In fact, vaccine hesitancy and antibiotic overuse frequently exist side-by-side, due to an imbalance in risk perception: some parents may overestimate the risk of rare vaccine adverse effects, while underestimating the adverse effects of antibiotic misuse and rising rates of AMR [35]. Improved (and equitable) immunization coverage can reduce antibiotic use, as has been evidenced in countries where the introduction of the pneumococcal conjugate vaccine has resulted in a decline in the prescribing of antibiotics for respiratory tract infections, and a reduction in the proportion of antibiotic-resistant invasive pneumococcal isolates [36].

### The role of climate change in propagating antimicrobial resistance in children in the Asia Pacific region

Climate change is a global health emergency that disproportionately impacts children, given their vulnerability to the illnesses propagated by extreme weather events and flooding [37,38]. This is particularly evident in the Asia Pacific region, where climactic warming is occurring more rapidly than the global average, resulting in increasingly frequent extreme weather events [38].

The predominant causes of death in children – respiratory tract infections, diarrhoea, malaria, and malnutrition – are all impacted by climate change [39], and as these conditions become increasingly common due to climate change, the widespread use of antibiotics to treat these conditions will drive further antibiotic selection pressure within the region [5].

Across the Asia Pacific region, climate change has already increased hospitalizations due to respiratory illness, gastrointestinal infections, and vector-borne diseases [40,41]. Arthropod-borne infections, particularly dengue and malaria, have increased in their prevalence due to the “extremely high” level of environmental shocks and stresses suffered by the region: between 2022 and 2023 alone, there was a 28% rise in dengue cases across the Pacific, which followed a ten-fold surge in cases globally from 2000 to 2019, due to increasing temperatures and higher rainfall and humidity [37,42]. Children presenting to hospital with dengue or malaria frequently receive empiric antibiotics to provide cover for the possibility of bacterial co-infection; and whilst co-infection can occur, overly broad-spectrum antibiotics are often prescribed for a prolonged duration, contributing to antibiotic overuse and propagating AMR [43].

Typhoid fever is one of the most common causes of bloodstream infections in children in the Asia Pacific region [7^▪^]. The bacteria that cause typhoid (enteric) fever – *Salmonella* Typhi – thrive in warmer temperatures, threatening the precarious gains made in reducing child mortality due to enteric fever, particularly with the emergence of antibiotic-resistant *Salmonella* spp. infections [44,45]. Over the last decade, the prevalence of antibiotic resistance against *Salmonella* spp. rose significantly in South Asia [46], accompanied by the emergence of extensively-drug resistant (XDR) *Salmonella* Typhi infections. First documented in Pakistan in 2016, XDR typhoid fever has now resulted in over 5000 infections globally, the majority of which have been diagnosed in children living in countries with limited access to clean water and sanitation [47]. Cases have been reported across many countries in Asia and also in travellers [48], and will continue to occur within the Asia Pacific region due to low typhoid vaccination coverage, rising temperatures and increasingly frequent flooding events.

### Challenges and progress in implementing antimicrobial stewardship and infection, prevention and control practices across the Asia Pacific region

**FIGURE 2:**
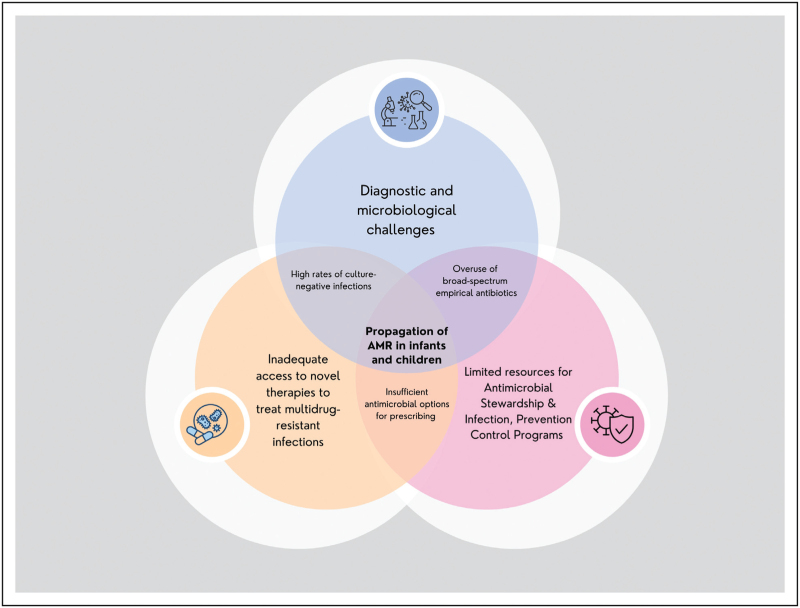
Intersecting challenges around diagnostic difficulties, insufficient resources for infection, prevention & control programs and limited antimicrobial stewardship propagates antimicrobial resistance in children in the Asia Pacific region.

Antibiotic overuse in children is common in the Asia Pacific region (as well as other resource-constrained healthcare settings), as access to microbiological diagnosis or even pathology tests indicating a potential bacterial infection, such as neutrophilia, are limited. Given the perceived vulnerability to infectious diseases, there is often community pressure for ready access to antibiotics, which has been perpetuated in many areas by limited and expensive healthcare access [49,50]. Consequently, implementing AMS programs to promote the judicious use of antimicrobials is challenging due to a complex interplay resulting in the propagation of antibiotic resistance mechanisms (Fig. 2).

**FIGURE 3:**
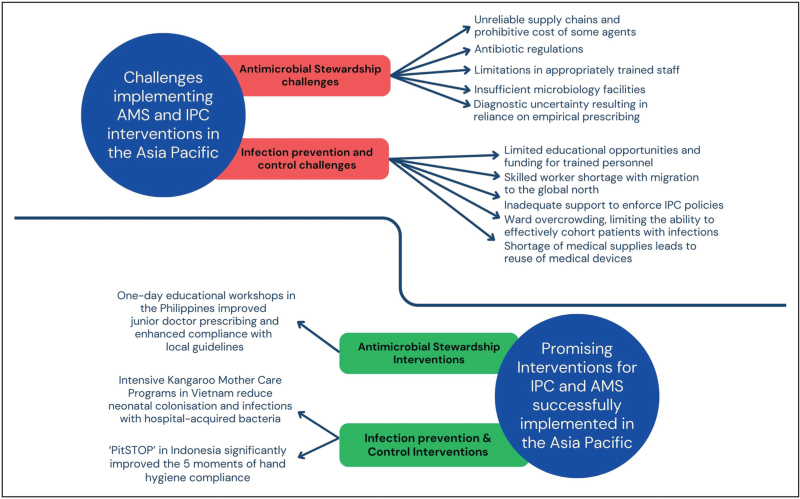
Many factors contribute to challenges in implementing AMS and IPC interventions in the Asia Pacific region; but local leadership has resulted in promising programs that have effectively reduced the burden of infection. AMS, antimicrobial stewardship; IPC, infection prevention and control.

In 2019, the WHO recommended the implementation of a continuum of care across diagnostic and antibiotic-prescribing stewardship programs in resource-constrained healthcare settings, alongside the rollout of multiple IPC strategies [51]. However, the implementation of AMS and IPC programs in these settings is challenging, hindered by both external and internal healthcare system barriers – such as supply chain issues, insufficient space to cohort or isolate patients with multidrug-resistant infections, and a lack of adequately resourced and trained staff (exacerbated by the Global North's poaching of skilled IPC professionals). Combined with resource constraints that often necessitate the re-use of medical devices and inadequate support (and prioritization of funding) to enforce AMS and IPC policies, many barriers to implementing effective AMS and IPC programs across the Asia Pacific exist (Fig. 3) [52,53].

Despite these challenges, there are success stories from across the region highlighting the potential for IPC and AMS programs to be implemented. In the Philippines, a one-day educational workshops for junior medical staff to reduce the over-prescribing of broad-spectrum antibiotics and encourage adherence to empiric treatment guidelines resulted in a successful change in antibiotic prescribing practices; while a nationwide media campaign highlighting the importance of prescription-only antibiotic use effectively raised awareness of AMR amongst the general population [9]. In Vietnam, greatly reduced antibiotic use has been demonstrated by implementing a simple algorithm for the management of childhood pneumonia, to assist clinicians to distinguish viral versus bacterial respiratory tract infections [54,55]. However, widespread implementation of these pragmatic models are often hindered by limited capacity to replicate research studies or alter policies given the many competing challenges clinicians face in the region, alongside limited assistance provided by existing funding models.

Replicating successful programs in other settings across the Asia Pacific should be a funding priority, spearheaded by regional leadership promoted to facilitate the implementation of similar strategies that promote the judicious use of antibiotics within both community and hospital settings across the region.

### Strategies to reduce the burden of antimicrobial resistance in children in the Asia Pacific

Reducing the current – and rising – burden of AMR in children across the Asia Pacific region will require a multifaceted and interdisciplinary approach, incorporating clinicians, researchers and policy-makers; working across healthcare, agriculture, and water and sanitation. Funding for improved surveillance programs that incorporate both microbiological and clinical data is necessary [56], alongside resources to strengthen developing IPC programs [9]. Enhanced access to improved diagnostic microbiology facilities is essential to promote the targeted use of efficacious antibiotics and implementation of AMS programs, which will be critical to implement to reduce antibiotic overuse across the region.

Concurrently, promoting vaccine development (and rollout) to reduce the infectious disease burden in children across the Asia Pacific is also necessary to reduce the rising prevalence of infectious diseases - particularly as climate change propagates this burden. Promisingly, vaccine development against multidrug-resistant gram-negative bacteria and Group B Streptococcus (GBS) have been established as a key international research priority, and once available, these vaccines may reduce the significant neonatal sepsis morbidity and mortality burden across the Asia Pacific (and globally) [57–60]. However, once developed, these novel vaccines will require international support to ensure equitable implementation across resource-constrained healthcare region, so that the benefits of vaccine development can reach the most vulnerable children [60]. Concurrently, funding should be prioritised to ensure all children across the Asia Pacific region can be immunized against pneumococcal disease to substantially reduce the burden of lower respiratory tract infections, one of the most common indications for antibiotic prescribing in children [61].

Alongside vaccine development and expanded uptake of available vaccines, promoting the development and availability of new antibiotics to treat multidrug-resistant infections in children is also urgently required. Although many novel agents have been developed and licensed to treat multidrug-resistant infections in adults, few have been licensed for use in children. Only 10% of antibiotics licensed for use since the year 2000 were made available to infants – and availability of these novel agents in children often lags accessibility in adults by at least a decade [24,62^▪^]. New antimicrobials need to be promptly licensed, and supported in their rollout via regional procurement and stewardship programs, to ensure multidrug-resistant infections can be effectively treated rather than resulting in high consequence outbreaks [63].

## CONCLUSION

Antimicrobial resistance is a ‘wicked’ global problem that is challenging to solve [3], and its effects are most acutely felt in settings such as the Asia Pacific - where multiple factors increase disease vulnerability, while concurrently there are insufficient resources to establish effective IPC and AMS programs. Most of the challenges highlighted in this review are established issues that require greater global solidarity and commitment to ensure better equity in vaccine and antibiotic access, both across regions and across age groups – particularly for vulnerable children. Local strategies have successfully been implemented to promote IPC and AMS programs, but their scale-up requires strong commitment from national governments across the Asia Pacific. Countries should be encouraged to have clear and actively implemented AMS and IPC programs, to document their challenges and successes, and to build a collective regional body of evidence to guide practice. Where best practice is identified this should be scaled up, with implementation research identifying the best local strategies to ensure sustainability.

## Acknowledgements

*None.*

### Financial support and sponsorship

*This study was supported by an Australian National Health and Medical Research Council (NHMRC) grant. The NHMRC had no involvement in the design or conduct of the research.*

### Conflicts of interest

*There are no conflicts of interest.*

## REFERENCES AND RECOMMENDED READING

Papers of particular interest, published within the annual period of review, have been highlighted as:

▪ of special interest
▪▪ of outstanding interest
